# Social norms in digital spaces: Conflict reports and implications for technology design in the teleworking context

**DOI:** 10.1007/s41449-022-00332-8

**Published:** 2022-12-01

**Authors:** Sarah Diefenbach

**Affiliations:** Department Psychologie, Ludwig-Maximilians-Universität München, Leopoldstraße 13, 80802 München, Germany

**Keywords:** Telework, Digital space, Digital communication, Home office, Wellbeing, Social norms, Technology design, Telearbeit, Digitale Räume, Digitale Kommunikation, Home-Office, Wohlbefinden, Soziale Normen, Technikgestaltung

## Abstract

With the increase of telework during the COVID-19 pandemic, employees’ interactions with colleagues have shifted almost exclusively to digital channels. Hence, it is critical to understand the underpinnings of successful collaboration and individual wellbeing in digital working structures. Combining qualitative experience reports and quantitative surveys from 145 teleworkers, this study sheds light on teleworking from a psychological perspective, taking social norms as a conceptual frame. The qualitative reports revealed five types of typical conflicts related to communication in the telework context, including both (1) technical problems (e.g., a bad connection) and psychological aspects such as (2) uncertainty or a lack of social feedback, (3) norm violations, (4) a lack of rules or meta-communication about appropriate behavior, and (5) digital communication barriers. Respondents’ quantitative ratings of qualities of telework versus working on-site revealed benefits of telework regarding task fulfillment and efficiency, but lower levels of motivation, conflict management, leadership, team spirit, inspiration and creativity. Participants qualitative reports on perceived challenges in remote work conditions included feelings of loneliness and increased demands related to self-management, creating boundaries between private and working life, motivation and self-regulation. This paper connects these findings with theoretical concepts from psychology and human-computer interaction and discusses implications for leadership and technology design.

*Practical Relevance*: This article discusses practical implications for leadership and technology design, e.g., interventions against conflicts in the context of digital work.

## Abstract (de)

Mit der Zunahme von Telearbeit im Zuge der COVID-19-Pandemie hat sich die Interaktion mit Kollegen im Arbeitskontext fast ausschließlich auf digitale Kanäle verlagert. Ein umfassendes Verständnis der Grundlagen erfolgreicher Zusammenarbeit und individuellen Wohlbefindens in digitalen Arbeitsstrukturen gewinnt somit zunehmende Relevanz. Die vorliegende Studie [*N* = 145] beleuchtet Telearbeit aus psychologischer Perspektive anhand einer Kombination von qualitativen Erfahrungsberichten und quantitativen Ratings und nutzt das Konzept sozialer Normen als konzeptionellen Rahmen. Eine Kategorisierung der qualitativen Konfliktberichte ergab fünf typische Konflikten im Zusammenhang mit der Kommunikation im Telearbeitskontext, darunter (1) technische Probleme (z. B. schlechte Verbindung) als auch psychologische Aspekte wie (2) Unsicherheit/fehlendes soziales Feedback, (3) Normverletzungen, (4) Mangel an Regeln und Metakommunikation über angemessenes Verhalten und (5) Kommunikationsbarrieren in digitalen Strukturen. Die quantitativen Ratings der Befragten Arbeitsqualitäten von Telearbeit im Vergleich zur Vor-Ort-Arbeit zeigten Vorteile von Telearbeit in Bezug auf Aufgabenerfüllung und Effizienz, jedoch ein geringeres Maß an Motivation, Konfliktmanagement, Führung, Teamgeist, Inspiration und Kreativität. Qualitative Berichte bezüglich wahrgenommener Herausforderungen der Home-Office-Situation bezogen sich auf Gefühle der Einsamkeit und erhöhte Anforderungen in Bezug auf Selbstmanagement, Abgrenzung zwischen Privat- und Arbeitsleben, Motivation und Selbstregulierung. Der vorliegende Beitrag diskutiert diese Ergebnisse in Verbindung mit theoretischen Konzepten aus Psychologie und Mensch-Computer-Interaktion und erörtert Implikationen für Führung und Technikgestaltung.

*Praktische Relevanz*: In diesem Artikel werden praktische Implikationen für Führung und Technologiegestaltung erörtert, z. B. Interventionen gegen Konflikte im Kontext der digitalen Arbeit.

## Introduction

Nowadays, a large share of our communication and interaction is mediated via digital technologies, especially in the work context. A large percentage of people worldwide engage in teleworking and at least sometimes use digital channels to collaborate with colleagues and customers. Broadly speaking, teleworking refers to working from home while communicating with one’s office by phone, email, or using the internet (Cambridge Dictionary 2021). Already in 2018, 70% of respondents in a global survey of over 18,000 professionals from a range of industries in 96 countries reported working outside one of their main company offices at some point during the week. 53% worked half the week or more outside the office (IWG 2018). In line with this, Mark Dixon, founder and CEO of the IWG International Workplace Group, declared flexible working to be the new norm (IWG 2019) and described how technological progress has fundamentally changed our perceptions concerning the locations and hours at which we work (Dixon 2018). Similarly, in his book on telework in the 21st century, Messenger (2019, p. 8) sees the current form of telework as a “whole new mode of work [which] has grown into almost every possible aspect of life” (Messenger 2019, p. 8).

In addition to technological advances and newly emerging opportunities, the relevance of telework has been further fueled by the need to replace physical meetings with digital structures whenever possible during the Covid-19 pandemic. In a global work-from-home experience survey conducted by Iometrics and Global Workplace Analytics (2020), 88% of respondents reported working from home regularly during the pandemic, meaning that telework became the standard method of communication and collaboration, at least for a period of time. For many employees, the complete shift to telework during the pandemic represented a completely new mode of work (Milasi et al. 2020) without much support to prepare for its specific challenges.

From a psychological perspective, one possible challenge of telework concerns the development of social norms and shared (implicit) communication rules as a basis for successful and fulfilling collaboration. When social interaction is transferred to a new (digital) context, there may be uncertainty as to which rules apply and to what degree norms from the non-digital world are transferrable (Diefenbach and Ullrich 2018). Misunderstandings and uncertainty about how to interpret co-workers’ behavior can lead to a negative team climate and increased conflicts, for example, if the other person’s behavior is interpreted as rudeness due to diverging ideas of social norms. If the change to a new social interaction context is quite rapid, as was the case during the Covid-19 pandemic, social conflicts due to misunderstandings and not yet established rules are even more likely. Making things even trickier, in the digital world, it is often not easy to detect whether a behavior perceived as negative is for technological reasons (e.g., the communication partner’s camera is switched off due to bandwidth limitations) or is the fault of one’s human counterpart (e.g., the communication partner doesn’t want to be seen/wants to eat during a meeting without the others knowing). While the relevance of social norms and frequently arising conflicts has already been discussed in different fields of HCI, the relevance of social norms in the telework context has not yet been discussed much or studied systematically. The present research aims to fill this gap.

Based on reports by 145 teleworkers collected in 2020, this research provides insights into daily experiences, misunderstandings and conflicts, needs and wants, as well as enhanced and endangered qualities of work in the context of digital communication and challenges specifically related to the working-from-home situation. Our research interprets and connects these with theoretical concepts from psychology and human-computer interaction (HCI), discusses potential implications for (digital) management and technology design, utilizing social norms as a conceptual frame. Before presenting the empirical study, the following sections describe our research objectives in more detail and briefly introduce relevant concepts and related research. The literature review first refers to wellbeing and social interaction in telework in general, then summarizes digital systems’ potential and challenges for remote collaboration with respect to communication qualities, and finally refers to social norm conflicts in digital collaboration and other fields of HCI, also highlighting research gaps with respect to social norms in the telework context.

## Research questions

The present research explores communication conflicts in the telework context, with a particular focus on social norms as a conception frame. While the primary goal is a deeper understanding of the different types of conflicts and the relevance of social norms within them, a subsidiary goal is to explore possible starting points for interventions to reduce such conflicts and whether the design of the technology itself could be a helpful starting point. As a first exploratory study, our research did not test specific hypotheses but was guided by the following sets of research questions:

- RQ 1—Conflicts and reflections on telework: What kinds of (social) conflicts emerge in teleworking communication? How do teleworkers experience interactions with colleagues over digital channels and how do they reflect on the working-from-home situation and its general challenges and advantages? Which are the main differences in perceived qualities of work between telework and working on-site?
- RQ 2—Social norms: What is the relevance of (a lack of) social norms and diverging interpretations of one another’s behavior for conflicts in teleworking communication? Do teleworkers differentiate between social norms for digital and non-digital meetings? Are social norms a helpful category to describe conflicts in the context of teleworking, after having already been applied in other contexts of HCI?
- RQ 3—Interventions and technology design: Which kind of interventions to prevent social conflicts in the telework context appear promising? Could the technology itself be a means to support shared social norms, and if so, what specific design influence do teleworkers appreciate?

Thus, RQ 1 takes a broad view, assesses the situation from a wider perspective, and aims to detect what is relevant for teleworkers. RQ 2 examines the working-from-home situation through a particular lens (i.e., social norms). Thereby, RQ1 and RQ2 tackle the issue from “two directions in parallel”. This was inspired by the qualitative research approach of combining model-based analysis and phenomenological insight (Diefenbach and Hassenzahl 2019). The rationale behind this method is that “models [here: social norms as a conceptual frame] will profit from deeper insights into constructs, their relevance and emergence in people’s everyday life, and, conversely, qualitatively described phenomena will benefit from model-guided integration [here: understand conflicts as social norm conflicts],” thereby “building a bridge between the exploratory and holistic potential of phenomenological data and the desire for integration and refinement in the context of a given model” (Diefenbach & Hassenzahl, p. 8). Finally, RQ 3 addresses the question of practical implications and possible interventions.

For each set of research questions, our research approach combines qualitative and quantitative approaches (see Table 1). Qualitatively categorizing responses to open-ended questions gives us a deeper understanding of teleworkers’ experiences, while quantitative ratings (e.g., positive and negative affect, the relevance of different qualities of work) help us determine the dimensions and relevance of the different issues.

**Table 1:** Overview of survey topics addressing to the research questions (RQ1–RQ3) Überblick der Fragebogenthemen und Bezug zu den Forschungsfragen (RQ1–RQ3)

RQ	Survey topic	Measure
1	Conflicts	Qualitative conflict report
		Quantitative rating of one’s own affect and assumed affective state of the other person involved in the conflict
	Working-from-home situation	Qualitative reports on perceived advantages and challenges
		Quantitative rating of liking the working-from-home situation at the moment and before the pandemic
	Qualities of work	Quantitative ratings of different qualities of work in telework versus working on-site
2	Social norms	Quantitative ratings of appropriateness of different behaviors in digital and non-digital meetings
3	Interventions and technology design	Qualitative report on ideas for resolving the conflict
		Quantitative ratings of different interventions to encourage positive norms in digital communication
		Quantitative ratings of different types of design influence

The first original contribution of the present study is to connect research on social norms in psychology and HCI with the telework context and explore to what degree this perspective can enrich prior theoretical understandings and possibly inform technology design. Our research introduces social norms lens as an insightful lens on HCI in the working context, that offers a deeper understanding of potentially relevant psychological mechanisms for wellbeing, conflict, and promising interventions in telework. While social norms have been highlighted as a source of conflict in other areas, they have not been investigated specifically in the telework context. The present study fills this gap. In addition, the study performs exploratory analyses of other potentially relevant factors of participants’ telework experience, such as their evaluation of the pandemic-induced working-from-home situation or comparisons of qualities of work in telework versus working on-site.

## Related work

The following sections summarize previous work related to our research questions such as studies on wellbeing and social interaction in telework (RQ 1), potential and challenges of digital systems for remote interaction (RQ 1, RQ 3) and social norms in digital collaboration (RQ 2).

### Wellbeing and social interaction in telework

Besides effects of telework on performance and productivity (e.g., Sánchez et al. 2007), researchers have shed light on the relationships between working from home and the facets of physical and psychological wellbeing related to a broad range of issues. This includes, for example, a study of mental health and stress symptoms among teleworkers compared to office-workers (Mann and Holdsworth 2003), consequences for organizational commitment, identification, and job satisfaction, team dynamics such as knowledge-sharing, or implications for work-family issues (Allen et al. 2015; Vittersø et al. 2003). Overall, the detected correlations and areas of risk can be considered highly dependent on the extent and specific design of telework. For example, Gajendran and Harrison (2007) found rather negative effects on relationships with coworkers when more than half of working hours were spent teleworking. Baruch (2000) reported that teleworking did not change conceptions of oneself as an employee as long as teleworking was balanced out by work in the ‘standard mode’. Later studies focused on specific mediators, identifying increased role ambiguity and reduced support and feedback as crucial for the negative effects of telework. Conversely, positive effects are related to increased autonomy (Gajendran and Harrison 2007; Sardeshmukh et al. 2012). Moreover, the quality of communication between team members may change in the context of telework. For example, contrasts of face-to-face and computer-mediated collaboration have revealed advantages for computer-mediated contexts regarding productivity, but a loss of socio-emotional content (Sauer et al. 2000). In sum, along the spectrum of qualities of work, from productivity and effectiveness on the one hand to experiencing team spirit, pleasure and work satisfaction on the other, the potential drawbacks of telework tend to concern the latter group of more experiential qualities (e.g., Baruch 2000; Mann and Holdsworth 2003; Sardeshmukh et al. 2012).

Going beyond direct effects of teleworking, researchers have also examined relevant contextual factors. In recent years, great emphasis has been placed on workplace culture as a crucial determinant of the success of flexible working arrangements (Abendroth and Reimann 2018). Based on linked employer–employee data for 4898 employees in large organizations in different industries, the authors conclude “how important it is to implement telework in a way that not only accommodates employers’ interest in flexibilization, but that it also makes it possible to reconcile work with a family life that involves high levels of responsibility” (Abendroth and Reimann 2018, p. 324). During the Covid-19 pandemic, integrating telework and private life became even more challenging, and digital culture, digital leadership concepts and team were found to be an essential element of responsible leadership. While earlier research on teleworking often focused on individuals working remotely or the effects of teleworking a few days a week (e.g., Baruch 2000), during the current period, working-from-home and existing as a team solely via digital structures has become standard. At the same time, the psychological side effects of telework (and digital collaboration in general) are becoming increasingly complex, requiring personal skills and resources far beyond basic skills such as time management (e.g., Baruch 2000; Beigi et al. 2018). As such, and although the many types of advanced videoconferencing software with numerous functionalities offer a multitude of opportunities, they also provide new space for misunderstandings and not-yet-established norms.

With regards to the present study focus, these previous findings on wellbeing and social interaction in telework are of relevance in several respects. First, previous findings show that employees’ wellbeing is dependent on the specific design of telework, highlighting the responsibility of employers and leadership to provide the best possible frame. This may include the used technology and introduced communication structure as well as aspects of team culture. As an advancement to previous research, the present study focuses on the particular role of social norms. Second, previous studies highlight the new challenges for the individual referring to the psychological side effects of telework such as dealing with (undetected) social norm conflicts among colleagues. Again, the pandemic context and sudden shift to a much higher share of telework makes research on how to support individuals’ wellbeing in the context of telework more timely than ever.

### Potential and challenges of digital systems for remote collaboration

From the first days of telework until now, researchers have explored to what degree technologies such as videoconferencing actually support effective, satisfactory collaboration between people communicating remotely (e.g., Biehl et al. 2015; Egido 1988; Olson and Olson 2000; Whittaker 1995). Typically, conversation quality over mediated channels is compared to face-to-face meetings, including studies of objective conversation characteristics, such as fewer contributions by individuals in remote compared to face-to-face conversations, difficulties noting peripheral cues, pointing to things or manipulating real-world objects, or having side conversations, (Isaacs and Tang 1993), as well as subjective measures, such as remote team members feeling less connected than their co-located peers (Biehl et al. 2015).

Other qualitative differences between remote and co-located collaboration may be harder for people to detect and verbalize. Even if a technology theoretically provides a satisfactory substitute for the information transfer required for a given task, in practice, people may still circumvent remote collaboration if possible—presumably because they do not experience the technology as a satisfactory substitute on a psychological level. For example, Olson and Olson (2000) reported that instead of using technology-mediated collaboration channels, people instead reorganized work so that they did not have to engage in close collaboration with a remote team member (Olson and Olson 2000). Similarly, Egido (1988) assumes that the main reasons why the uptake of videoconferencing in the 1980s remained below expectations lie beyond the scope of technological and economic analysis, and instead concern psychological and sociological factors, such as reduced opportunities for informal interactions. Indeed, many of the envisioned use cases and associated reductions in traveling costs were not realized. As Egido (1988, pp. 18/19) argues, the fact that “it is often over informal chats outside of official meeting rooms that important information is transmitted and real decisions are made” (e.g., Mintzberg 1973) “makes teleconferencing unattractive to politically savvy employees”.

Since then, many additions to standard videoconferencing, such as chat, polling tools, and interactive (online) whiteboards have been developed and have become a routine form of communication for teleworkers. Simultaneously HCI researchers have tried to address the shortcomings of traditional remote technology and have developed design solutions to meet specific requirements. One such path is designing different types of embodied telepresence devices that seek to mitigate the social disadvantages of video-based communication by providing remote users with a local embodiment, ranging from fully mobile robots to smaller devices that sit on a meeting room table (Biehl et al. 2015). Ideally, such an embodied representation of a remote person will establish a reciprocal sense of “being there” and harmonize interaction between remote and co-located team members. Indeed, Biehl et al. (2015) found that local participants interacted with a remote participant in a fashion more similar to face-to-face when using an embodied technology—potentially due to an increased perception of the remote person’s agency.

A technically even more technologically advanced variant of telepresence is the Room2Room system and its life-size virtual projections of the remote person (Pejsa et al. 2016), which is intended to create an illusion of the remote person’s physical presence in the local space and support a shared understanding of verbal and non-verbal cues (e.g., gaze, pointing) In their evaluation study, Pejsa et al. (2016) compared their Room2Room system to the established teleconferencing system Skype. While using the Room2Room system led to an enhanced feeling of the communication partner’s presence, participants did not have an opportunity to see what their communication partner was seeing. While the Skype interface shows the view from both the current user’s camera and their conversation partner’s camera, the Room2Room system lacks such a feature. This in turn makes it more difficult to gauge projection quality on the other end and assess whether one’s communication partner can see one’s nonverbal cues (Pejsa et al. 2016, p. 1723). This example shows that the limitations and also specific psychological benefits of such systems often first become apparent in the actual usage situation, and that the requirements for satisfactory communication can be difficult to foresee.

In sum, despite recent technological advances as virtual reality projections, communication and collaboration over mediated channels will always have some limitations, and distance still matters (Olson and Olson 2000). As outlined by Olson and Olson (2000), this becomes visible when considering many basic characteristics of co-located interaction that affect communication quality. These include “shared local context”, i.e., participants experiencing similar conditions regarding the time of day or local events, which provides a framework a frame for easy socializing and a mutual understanding of what is on the other person’s mind. “Informal hall time before and after” is a basis for opportunistic information exchange and social bonding. “Personal information” means that contributors’ identities are usually known and can be taken into account. In sum, the full list of subtle but meaningful qualities of co-located interactions demonstrates how difficult it is to simulate such communication such communication via mediated channels and to establish what Olson and Olson (2000) refer to as “common ground” (Clark 1996). Common ground refers to the knowledge that communication partners have in common and are aware that they have in common. As laid out by Olson and Olson (2000, p. 157), we typically have specific assumptions about what different people know and use these to frame what we say: “People describe the same event or idea quite differently talking to a spouse, a coworker, a distant relative, a neighbor, a stranger from across the country, and a stranger from overseas […] We establish common ground not just from some general knowledge about the person’s background but also through specific knowledge gleaned from the person’s appearance and behavior during the conversational interaction itself. If we say something based on an assumption about what someone knows, but their facial expression or verbal reply indicates that they did not understand us, we will revise our assumptions about what common ground we share and say something to repair the misunderstanding”.

In remote communication, this quality of common ground, and hence opportunity to identify and repair misunderstanding, is limited. Various communication cues are missing depending on the channel. The fewer cues there are, the more likely misinterpretations become—and the conversation might move on before the conversation partners are able to reach correspondence (Olson and Olson 2000, p. 158).

Although various approaches to enriching digital communication with more communication cues exist (e.g., emoticons in chat conversation), such design “solutions” can also lead to more misunderstandings (e.g., Miller et al. 2016). Often, there is no common ground on how to use and interpret such communication cues in the digital realm, as they are usually expressed spontaneously and unconsciously in the face-to-face context. Already in the 1990s, Isaac and Tang (1993) described the problems that arise if a videoconferencing system forces people to take explicit actions to engage in behaviors that normally take place unconsciously. Moreover, artificial behaviors may be interpreted differently by other participants: “For instance, a person who would have been seen as enthusiastic might be perceived as dominating if she uses an explicit mechanism [here: a visual mechanism] rather than a socially negotiated one to manage floor control” (Isaacs and Tang 1993, pp. 204/205). Due to the limited opportunities to apply social norms from face-to-face conversation, technology-mediated meetings often include a lot of organizational speech rather than real, content-related communication, people develop behaviors to compensate for shortcomings (e.g., always identifying oneself before speaking, more formal protocols for turn-taking), and more explicit discourse rules are needed (Olson and Olson 2000, p. 153).

In sum, the studies cited above reveal a strive for ever better and more advanced technical tools for remote collaboration, but also still existing pitfalls and potential for interpersonal conflict. Though not focused explicitly, the lack of shared social norms and discourse rules appears to be a recurring issue in many of these studies. Our research picks up this subject for deeper analysis and further connects it to interventions and technology design. In the following paragraphs, we summarize previous studies on social norms in digital collaboration and other fields of HCI beyond the work context.

### Social norms in digital collaboration and other fields of HCI

Social norms are informal understandings that govern the behavior of societal members, i.e., shared beliefs regarding appropriate ways to feel, think and behave (Turner 1991). Social norms help us to interpret others’ behavior and react appropriately (Horne 2001). In contrast to legal norms (e.g., laws), which are created in a deliberate process, set down in written tests, and linked to specific sanctions and enforcement mechanisms, social norms occur spontaneously rather than being planned deliberately, usually remain unwritten, and are enforced informally (Hechter and Opp 2001). Therefore, we are usually not aware of the many unwritten laws underlying our behavior, which only become evident when conflict arises: if someone behaves in a way that contradicts our informal understanding of what is appropriate, such as cutting in line, entering an office without knocking, or starting to eat before everyone is seated at the table. Since the beginning of mankind, such norms have naturally developed from interpersonal interactions (Horne 2001; Popa et al. 2014), although the typical contexts of social interaction have changed over time—from gathering around the hearth to today’s meetings in chatrooms and other digital spaces. Understandably, whenever a new social interaction context emerges, conflicts may occur in the beginning, since established norms do not yet exist and people are unsure which norms may be transferrable from other contexts (Diefenbach and Ullrich 2018).

Accordingly, as communicating via email and the Internet became increasingly common in the work environment, many conflicts and confusion around social norms for communication technologies arose, and researchers debated the need for “digital etiquette” (e.g., Preece 2004). Indeed, no shared norms seem to have been established yet even with respect to basic questions such as the preferred salutation in email communication. This was illustrated by Diefenbach and Ullrich (2018, p. 51 ff.), who highlighted contrasting recommendations about appropriate email salutations: An interviewee in the study by Preece (2004, p. 58) clearly stated that “… not addressing me by my name and ending without a farewell greeting and the sender’s name—that’s rude and unfriendly”. Later on, an online discussion around the question “How do you address someone in your reply to their emails?” (Ars Technica Open Forum 2014) showed the norm of addressing people by name in email is quite controversial, with vastly different reasons given for (not) addressing others by name in email communication. For example, one user sees addressing someone by name as an anachronism, stating that “No name whatsoever. I just reply without any kind of anachronistic salutation line. Death to the letter and everything it stood for.” Another user emphasizes on how not addressing one’s counterpart by name might even be a sign of valuation and intimacy: “In personal email that’s a reply, I often don’t even use the name. You’ve emailed me. You know me. I know you.” In a similar vein, another user draws a parallel to face-to-face conversation: “I don’t think I have ever put a formal greeting in an email, even back in olden times. Of course, I don’t use someone’s name when talking to them in person either, they know who they are, no need to remind them.” Finally, another forum user comments that “It depends entirely upon the relationship and what kind of email”, i.e., there are no general rules. Hence, what one person experiences as rudeness could actually be a sign of friendship for another (Diefenbach and Ullrich 2018, p. 51).

Beyond the email context, numerous studies have explored the evolution of social rules in digital spaces and newly emerging communication channels such as social media (e.g., Moncur et al. 2016; Sabra 2017; Voggeser et al. 2018), videoconferencing and systems for mobile remote presence in the workplace (Lee and Takayama 2011), chat tools in online libraries (Radford 2006), online gaming (Martey and Stromer-Galley 2007), or online communities (Kim 2000). Also, researchers have examined to what degree social norms from interpersonal interaction (e.g., eye contact) are transferred to digital environments such as videoconferencing (Yee et al. 2007), as well as social dilemmas resulting from mixed contexts and dealing with social norms from the physical and virtual contexts at the same time (e.g., answering mobile calls in various circumstances (Inbar et al. 2014)). In sum, universally shared norms of social interaction in digital spaces are rare, with divergences in norms often associated with different sub-cultures or user groups, e.g., between online library employees and customers (Radford 2006).

Thus, in many digital contexts, there still is no consensus about what is right or wrong and appropriate or inappropriate behavior (Diefenbach and Ullrich 2018; Tene and Polonetsky 2014). According to Tene and Polonetsky (2014, p. 60), with the new types of socialization resulting from technological innovation, we often have “nothing more than a fleeting intuition as to what is right or wrong” and “intuitions and perceptions of how our social values should align with our technological capabilities are highly subjective”, driving us into a “techno-social chaos” (Tene and Polonetsky 2014, p. 60). As described by Diefenbach and Ullrich (2018, p. 44 ff.), the gradual development of routines around technology use, particularly in the field of communication technology, often breaks up such established social norms. A typical example is the commitment to fixed appointments (old norm) versus cancelling or delaying an appointment at the last minute, which can be easily done nowadays via text message. While the change and evolution of social norms over time is a natural process, the speed of this change has increased dramatically. In many cases, the speed of technological progress may exceed the tempo at which a society typically develops consensus about the informal rules governing societal life.

From this perspective, a lack of social norms could also be a risk for digital communication in the telework context, potentially harming relationships between colleagues, teams and customers due to the lack of a feeling of unity. While social norms and shared standards for social interaction have been acknowledged as crucial for team spirit and work motivation (e.g., Ehrhart and Naumann 2004; Ellemers et al. 2004), there are a lack of studies that consider this issue in light of our increasingly digital communication structures, such as in the context of telework. Shared norms can form a foundation for cohesion and positive social interaction experiences in the digital space as well (e.g., Diefenbach and Ullrich 2018; Hong et al. 2019). In this sense, social norms can be considered a potential source of conflict, but also an important resource in the context of telework. Accordingly, a particularly interesting question is to what degree technology itself could support positive or shared norms. First experiments on how technology could support shared communication rules and other behavioral norms in daily life include different fields, such as norms of punctuality when meeting friends (Diefenbach et al. 2017; Diefenbach and Ullrich 2018), norms of salutation in online forums (Mooseder 2018), or norms in mobile phone communication (Inbar et al. 2014).

With regards to the present study and its advancements to previous research, the above cited examples suggest social norms as a promising starting point to understand and improve digital interaction. However, so far, this has rarely been investigated in the telework context, where in fact the business software used could be an interesting resource and starting point for interventions. Therefore, in this survey of teleworkers, a particular focus was placed on social norms, providing a conceptual frame to understand the dynamics of conflict in digital communication in the telework context. The next sections present the survey and questions that were used to gain deeper insights into employees’ daily life experiences in the context of digital communication and the pandemic-induced working-from-home situation.

## Materials and methods

In order to gain insights into the experience of telework, related conflicts and wishes, potentials for improvements through technology design, and teleworkers’ evaluation of the working-from-home situation we conducted an online survey using the survey software SurveyMonkey (surveymonkey.com).

### Survey

The survey began with a short introduction to the study topic, information about data processing and privacy, and asking participants to provide their informed consent to participate in the study. After this, as summarized in Table 1, the survey covered different subtopics and measures referring to our three research questions on digital communication and the telework context. In addition, we assessed demographic data and characteristics of telework (i.e., technology used, frequency of telework). The following sections describe the applied measures in detail.

#### Conflicts

Participants were asked to report on a recent digital communication situation in the work context they had experienced as irritating or conflict-laden. In addition to providing a qualitative report on the situation, participants also named the technology and other person(s) involved. Additionally, participants rated their own affect as well as the assumed affective state of the other person in the described situation. This allowed us to compare own-other affect ratings and assume typical conflict dynamics such as competitive victimhood and escalating processes (Diefenbach and Ullrich 2018; Noor et al. 2012). Affect ratings were assessed on a five-point scale (1 = negative, 5 = positive).

#### Working-from-home situation

Beyond the issue of digital collaboration, participants further described their experiences concerning the current working-from-home situation, namely working and living at the same place, with different measures: respondents provided an overall evaluation of how much they liked working from home at the moment and before the pandemic on a five-point scale (1 = not at all, 5 = very much). Perceived major advantages and challenges of working from home were captured with open-ended questions to provide space for respondents to emphasize the issues most personally relevant to them.

#### Qualities of work

The survey also assessed whether participants felt that different qualities of work (e.g., task fulfillment and efficiency, pleasure and motivation, problem-solving and conflict management, leadership, team spirit, inspiration and creativity) are better fulfilled through telework or working on site. The surveyed qualities captured dimensions of classical theories of work satisfaction and job design, such as “quality of supervision” and “relations with others” in Herzberg’s (1974) two-factor theory, or “feelings of achievement and task accomplishment” and “social interaction and team work” in the motivational job design approach by Campion and Thayer (1987). Moreover, we considered research on employee needs and incentives (e.g., Remer 1978), which refers to work task-related needs (e.g., room for autonomy and creativity) as well as social interaction (e.g., opportunities for problem-solving conversation; opportunities for conversation with colleagues). Here as well, participants provided ratings on a five-point scale (1 = definitely better in telework, 2 = a bit better in telework, 3 = no difference, 4 = a bit better when working on-site, 5 = definitively better when working on-site).

#### Social norms

Social norms in digital and non-digital meetings were assessed by having participants rate eight distinctive behaviors as appropriate or inappropriate in each context on a five-point scale (1 = inappropriate, 5 = appropriate). The surveyed behaviors were: (1) temporarily leaving the room without giving a reason (e.g., to go to the bathroom), (2) drinking during the meeting, (3) eating during the meeting, (4) texting on one’s smartphone, (5) , checking emails, (6) averting one’s gaze (from the screen/from others), (7) taking notes on a laptop, (8) taking notes by hand. The list of surveyed behaviors was based on typical aspects of (digital) meeting etiquette in the literature and blog posts (e.g., Browne 2020; Driska and Reid 2020; Schaffner 2008; Williams-Jones 2020), as well as informal brainstorming in the research team.

#### Interventions and technology design

This part of the survey dealt with how different kinds of interventions could establish and support appropriate behavior and positive norms in digital communication. Positive norms refer to basic behavioral rules that are seen as beneficial for the team and thus will hopefully be taken up by team members (e.g., punctuality, let others finish before beginning speaking). First, participants jotted down their ideas as to a possible intervention that could have helped prevent the conflict they described earlier in an open-ended answer format. In addition, the participants’ views on a set of predefined interventions to develop positive norms in digital communication were assessed on a five-point scale (1 = not agree at all, 5 = totally agree). These predefined interventions proceeded from four different starting points:

1. leadership—rules are specified by a leader (e.g., supervisor, team coordinator)
2. meta-communication—rules are discussed and specified together as a team
3. personal responsibility—providing guidelines to support self-reflection and positive norms (e.g., what impression do I make on others? what norms do I communicate through my behavior?)
4. technology design—the technology itself is designed in a way that makes committing to basic team rules more likely (e.g., punctuality).

This set of four starting points was inspired by common intervention approaches discussed in the occupational health and workplace health promotion literature (e.g., Sánchez et al. 2007), which stresses multiple actions at various levels (e.g., individual, interpersonal, organizational). For example, the desired behavior (change) can be supported through leadership and organizational rules, employee participation in workshops and meta-communication on a team level, but by equipping employees on the individual level equipped with trainings or other material to enhance relevant knowledge and competencies. In addition, technology design was considered as aa forth starting point for establishing the desired behavior (change), informed by the approach of transformational products and persuasive technologies approach (e.g., Fogg 2003; Laschke et al. 2011). Examples of persuasive design concepts in the work domain from previous research cover a wide field of applications and behavioral goals, such as sustainable behavior at the workplace (e.g., Lockton et al. 2014), motivating employees in the health sector to improve their habits of recording patient sessions (Selassie et al. 2017), or a positive work attitude and commitment to the organization’s ideals, visions and mission (Nkwo 2019).

Given that the specific design influence strategy adopted can be decisive for the effectiveness and acceptance of a technology (e.g., Selassie et al. 2017), we were interested in which type of influence might be best-suited for promoting shared norms at the workplace. Accordingly, our survey explored concrete design solutions within the design influence classification scheme by Tromp et al. (2011), which systematically compares different types of influence. This classification scheme was originally introduced in the field of designing for socially responsible behavior (e.g., a camera that detects speeding to encourage responsible driving) and classifies different types of influence along two dimensions: hidden vs. apparent and weak vs. strong, resulting in four unique types (i.e., decisive, coercive, seductive, persuasive). Coercive design, for example, is strong and apparent in its influence; an example would be a camera to detect speeding. In contrast, seductive design is weak and more hidden/implicit in its influence; an example would be a poster campaign that seeks to encourage responsible driving through messages such as “watch your speed”.

Transferring this concept to the field of social norms in digital communication, participants were asked how exactly technology should support commitment to team rules such as punctuality. Each option represented one type of design influence:

Technology should be designed in a way that …

- … encourages commitment to rules such as timelines, e.g., a videoconferencing system that encourages timelines by notifying users of how long the others are already waiting when dialing in [apparent, weak = persuasive influence]
- … enforces commitment to rules such as punctuality, e.g., a videoconferencing system where it is no longer possible to dial in when more than two minutes late [apparent, strong = coercive influence]
- … supports commitment to rules such as punctuality in a gentle way, e.g., a videoconferencing system that displays each participant’s time of entry [hidden, weak = seductive influence]
- … supports commitment to rules such as punctuality in a decisive way, e.g., a videoconferencing system that mutes participants who are late for the first two minutes to not disrupt the current flow of communication [hidden, strong = decisive influence]

Participants stated their level of agreement with the different options on a five-point scale (1 = do not agree at all, 5 = totally agree).

#### Demographic data and telework characteristics

Demographic information included gender, age, number of household members, occupation and industry as well as use of communication tools, videoconferencing software and groupware in the work context. The frequency of telework at the moment and before the pandemic was assessed on a five-point ordinal scale (1 = never, 2 = occasionally/max once a week, 3 = frequently/1–2 times a week, 4 = most of the time/3–4 times a week, 5 = all the time).

### Procedure

The data were collected in Germany via an online survey over a four-week period in July/August 2020. The study was presented as a survey of digital communication in the work context and the study invitation was disseminated via email to two different research panels as well as snowballing to friends and acquaintances. The only inclusion criterion for participation was use of digital communication in the work (or university) context. The survey took about 15 min to complete. Four 50 € Amazon vouchers were raffled off among all participants as an incentive for participation. In line with the APA’s ethical principles for psychologists, the Declaration of Helsinki, code of conduct and university ethical review board standards, participants provided their informed consent to participate in the study before starting the survey. Data was processed and recorded according to the DGSVO and the according recommendations of the German Psychological Society’s (DGPs).

### Sample

277 individuals started the survey, of whom 145 (45%) completed it and thus constitute the sample for the present analysis. The participants were between 18 and 64 years of age (M = 32.41, SD = 11.10); 92 (63%) indicated their sex as female, 53 (37%) as male, with diverse professional backgrounds, such as engineering, business consulting, education, medicine and health, information technology, media and publishing, administration and law, or the public sector. The majority were regular employees. Some were self-employed or worked as paid student interns.

### Analytic strategy

In addition to descriptive analyses of the survey questions, for some questions, t‑tests against the scale midpoint were performed to assess the participants’ decisiveness, and pairwise t‑tests were employed to test interesting comparisons. Differences are reported as significant with an alpha level of < 0.05. Because multiple comparisons were conducted, the results were adjusted for alpha error accumulation using Bonferroni corrections. The software SPSS was used for statistical analyses. Effect sizes (Cohen’s d) were calculated with the online software StatistikGuru (Hemmerich 2015, 2020). The qualitative data from the open-ended questions were coded into categories. The categories were created by an independent rater (a psychologist trained in qualitative data analysis). A second rater (also a psychologist trained in qualitative content analysis) classified the open-ended responses based on this categorization scheme. The interrater agreement was satisfactory, with Cohen’s Kappa values for each question ≥ 0.82.

## Results

**Fig. 1:**
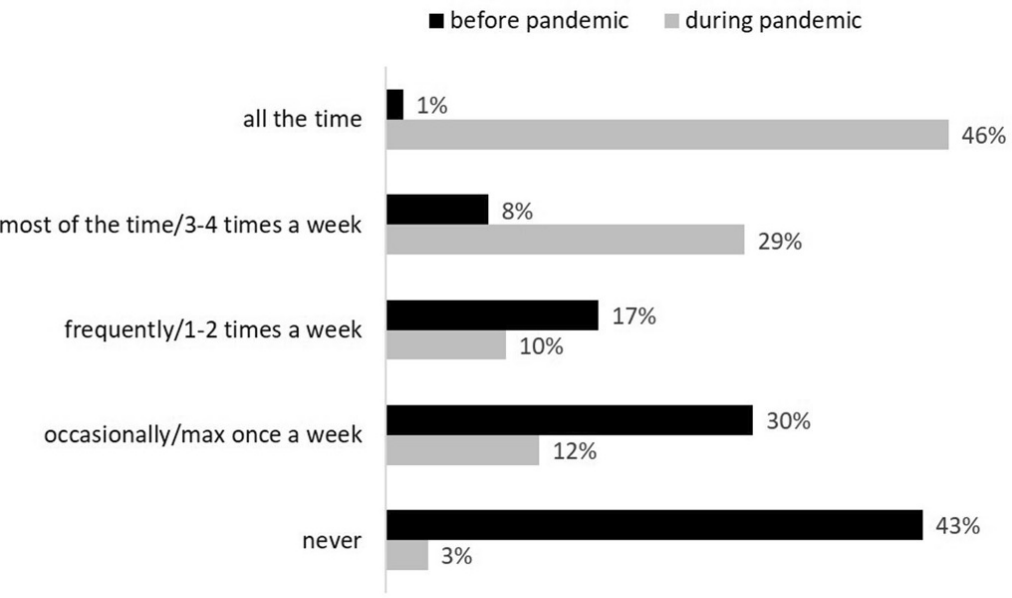
Frequency of telework during and before the pandemic [*N* = 145] Häufigkeit von Telearbeit während und vor der Pandemie

The following sections present our survey findings clustered along the three sets of research questions on telework. In addition, Fig. 1 shows the frequency of telework currently and before the pandemic. More than 70% had never or only occasionally worked from home before the pandemic, whereas now more than 70% teleworked most or even all of the time. Thus, before the pandemic, many people never experienced telework, and during the pandemic, they had their first telework experience.

### RQ 1—Conflicts and reflections

#### Conflict reports

Participants’ qualitative conflict reports revealed a variety of different types of conflicts. In addition, differences in content, the described situations could also be differentiated based on the other person(s) and the technology involved. To provide a broad initial overview, the conflict descriptions were broadly categorized according to these three criteria (content, persons, technology).

Tables 2, 3 and 4 show the frequencies with which each category of conflict was mentioned, with respect to the involved persons, technology and content. In many cases, the involved persons were colleagues (or classmates in the case of university students). Fewer participants reported conflicts with customers or principals. In most cases, the conflicts occurred in the context of videoconferencing, with Zoom and Microsoft Teams being the most frequently mentioned software (although some participants did not report the specific software used).

**Table 2:** Conflict characteristics: Categories of involved person(s) and relative frequency of mentions [*N* = 145] Konfliktcharakteristika: Beteiligter Person/en (Kategorisierung) und Häufigkeit der Nennungen [*N* = 145]

Category of involved person(s)	Frequency of mentions (%)
Colleagues	41
Colleagues and boss/supervisor	28
Principal	6
Customer	6
Other	19

**Table 3:** Conflict characteristics: Involved technologies and relative frequency of mentions [*N* = 145] Konfliktcharakteristika: Beteiligte Technologie (Kategorisierung) und Häufigkeit der Nennungen [*N* = 145]

Involved technology	Frequency of mentions (%)
Zoom	43
Microsoft Teams	10
Skype	5
WebEx	3
Slack	1
WhatsApp	1
Email	1
Videoconferencing software (not further specified)	15
Mobile device (not further specified)	6
Other	11

**Table 4:** Conflict characteristics: Categories of conflict content and relative frequency of mentions [*N* = 145] Konfliktcharakteristika: Inhaltskategorien und Häufigkeit der Nennungen [*N* = 145]

Category of conflict content	Frequency of mentions (%)
Technical problems	32
Uncertainty, misunderstandings, lack of social feedback	23
Norm violations	8
Lack of rules	11
Communication barriers in digital structures	7
Other	19

Participants’ quantitative ratings of the experienced affect in the reported situation were rather negative (M = 2.20, SD = 0.94), and significantly lower than the scale midpoint (t (144) = 10.17, *p* < 0.001, d = 0.84). The assumed affective state of the other person involved was also rated as rather negative (M = 2.62, SD = 9.35), albeit significantly more positive than the participants’ own feelings (t (144) = 4.79, *p* < 0.001, d = 0.40). This indicates that the reported conflict was perceived as more negative for oneself than for the other person. Thus, the participants tended to believe that they suffered more from and were the “true” victim of the conflict.

In terms of content, five broad types of conflicts could be identified from the qualitative reports, as further illustrated by concrete examples.

One category comprised (1) **primarily technical problems** (e.g., bad internet connection), often related to delays and negative impressions (e.g., being late because of problems dialing in). Typical quotes were:

“There was a delay in audio transfer. Accordingly, we both talked at the same time and interrupted each other.” [P53]

“I had technical problems during a Zoom meeting. My computer logged me out. This totally destroyed the work flow. It was quite embarrassing for me.” [P134]

Another category comprised reports on (2) **uncertainty, misunderstandings, or a lack of social feedback** caused by technical barriers. For example, one participant described difficulties adequately assessing a customer’s reaction in a video call due to background noise and the limited possibilities to assess nonverbal communication cues. Similarly, several participants felt irritated by the lack of feedback and encouragement from the audience when speaking in a meeting, especially when others had turned off their cameras. As one participant described:

“You see only black boxes, you are talking into emptiness” [P20]

Also, participants described situations of uncertainty about whether a colleague’s lack of reaction reflects disinterest or technical problems. Some even assumed that others “fake” technical problems for reasons of convenience (e.g., switching off one’s camera to do other things in parallel, faking audio problems to avoid an oral quiz), which negatively impacted the atmosphere and team spirit. For example, P138 described:

“The weekly physics seminar was held via Zoom and started with an oral test. In retrospect, it turned out that some students faked audio problems to avoid the test situation” [P138]

Overall, many participants experienced the digital meeting as “not real communication”, especially when communication was split over different software channels. For example, one participant described a seminar situation where the instructor used the audio channel (speaking), whereas the seminar participants refused to turn on their cameras or audio and reduced their communication to chat:

“This felt very strange, as if the instructor was talking to himself. I wonder how he might feel when he is leading us through the whole seminar but never gets any feedback from our side. Of course I also feel sorry for him—but if nobody is talking I also don’t want to start. I at least posted a little thank you in the chat at the end of the seminar.” [P86]

Another category of conflict reports referred to (3) **norm violations** and (in the respondents’ opinion) rude behavior, such as eating during a video call, leaving the room without notification, interrupting others, endless monologues without acknowledging others’ requests to speak, and limited awareness of one’s own impression on others. In some cases, participants explicitly discussed differences between the experienced situation and a corresponding non-digital meeting. For example, one participant complained:

“Two colleagues joined a digital meeting from one desk and were constantly whispering with each other with their microphone muted. This gave the impression that they were gossiping about the others. Presumably, they would not have done this in the same obvious way during a face-to-face meeting.” [P89]

Likewise, another participant (P92) complained about her classmates undermining a Zoom lecture:

“In an online Zoom lecture, the professor’s cat entered the picture and then rested on the professor’s lap. The students started an extensive chat discussion about the cat, often just silly comments like ‘meow!’. The professor thought the incoming comments were referring to her lecture, was continuously distracted and had serious problems continuing with her regular lecture content. I found my classmates’ behavior irritating and dismissive. I suspect that in a physical meeting, they would have stopped earlier and realized that the joke is over” [P92]

Somewhat related to the former, another category addressed a (4) **lack of rules** or meta-communication about appropriate behavior, e.g., a lack of agreement on how to “raise your hand” in digital meetings or frustration about colleagues’ neglecting (seemingly self-evident) rules such as muting their microphone when not speaking, especially when background noise is present (e.g., the co-worker’s wife working on a sewing machine, P43). Due to this lack of communication rules, meetings often took more time than necessary—even with respect to simple procedures like a round of introductions:

“At the beginning of a videoconference we were asked to introduce ourselves. However, there was no fixed order of speakers. It took ages until someone started speaking. Always irritating pauses; nobody wanted to interrupt. Actually I introduced myself last, just to be sure I do not interrupt anyone.” [P72]

A fifth category (5) referred to general **communication barriers **or pitfalls in digital structures, e.g., figuring out who is speaking when you do not know the other participants’ (voices) very well, and the generally higher attentional demands compared to face-to-face settings. A typical quote was:

“Too many participants who do not know each other personally. Matching voices and names is almost impossible, especially if multiple persons log in from one account.” [P12]

Participants also referred to difficulties in initiating small talk (e.g., in a waiting situation) because unmuting one’s microphone is a barrier. Also, there is less room for informal half-private conversations between two persons, as typically initiated at the beginning or end of a (non-digital) meeting. Some participants also described difficulties in mixed meeting contexts, e.g., everyone else being physically present in a room and one person joining via Zoom, stating that communication would have been easier and more inclusive if all participants had connected via Zoom.

Finally, a final category (6) comprised **other** types of conflicts with no (obvious) relation to the digital context, e.g., inadequate comments by colleagues.

#### Evaluation of working from home

The respondents’ quantitative evaluations of working from home currently were in the upper range (M = 4.00; SD = 1.70) of the applied five-point scale (liking, 1 = not at all, 5 = very much) and significantly higher than their (retrospective) evaluations of working from home before the pandemic (M = 1.94, SD = 1.03; t (143) = 21.15, *p* < 0.001, d = 1.76). Tables 5 and 6 show the categories built from the participants’ qualitative responses concerning perceived challenges and advantages of the working-from-home situation. Note that because some participants declared that they did not see any challenges (or advantages) related to the working-from-home situation, the samples employed for categorization are somewhat reduced, with *N* = 136 naming challenges and *N* = 142 naming advantages.

**Table 5:** Perceived challenges in the working-from-home situation [*N* = 136, multiple mentions possible] Wahrgenommene Herausforderungen der Home-Office-Situation [*N* = 136, Mehrfachnennungen möglich]

Categories of challenges in the working-from-home situation	Frequency of mentions (%)
Isolation/lack of social interaction	40
Lack of equipment, ergonomic problems	18
Boundary management	12
Motivational problems/self-regulation	8
Distractions/concentration problems	15
Other	7

**Table 6:** Perceived advantages of the working-from-home situation [*N* = 142, multiple mentions possible] Wahrgenommene Vorteile der Home-Office-Situation [*N* = 142, Mehrfachnennungen möglich]

Categories of advantages of the working-from-home situation	Frequency of mentions (%)
Flexibility/individual time management	37
No commute	53
Fewer distractions/more concentrated work	4
Better wellbeing, due to healthy food, comfortable clothes, etc	3
Other	3

Among the perceived advantages, the most frequent statement concerns the lack of commute and associated gain in free time, followed by the enhanced flexibility and opportunities for individualized time management. 90% of statements primarily related to one of these two categories. A few other participants mentioned fewer distractions from colleagues, and thus more concentrated work as the main advantage of working from home. Some referred to better physical wellbeing, e.g., due to wearing more comfortable clothes and better food than in the office.

Among the perceived challenges, a major category concerned isolation and the lack of social interaction with colleagues. In particular, participants reported missing natural occasions for small talk and casual information exchange, thus losing the feeling of “knowing what is going on”, which impairs feelings of relatedness and security.

Another frequently mentioned category concerned a lack of (technical) equipment and related ergonomic problems (e.g., cannot sit in an upright position because one is working with a laptop only and no external monitor). In addition, another sizable share of participants named distractions or concentration problems in the home setting as the main challenge of working from home, and as a somewhat related category, problems managing the boundaries between private and working life. As one participant stated:

“It is quite hard to communicate to family members that I am physically sitting here but not available, namely, ‘not here’” [P114]

A final category of challenges concerned motivational problems and an increased need for self-regulation and self-discipline. A typical quote was (P94):

“Back then, when working on-site, I only needed to motivate myself to work once in the morning—when working from home, I have to do this all day long” [P94]

#### Qualities of work in telework versus working on site

**Fig. 2:**
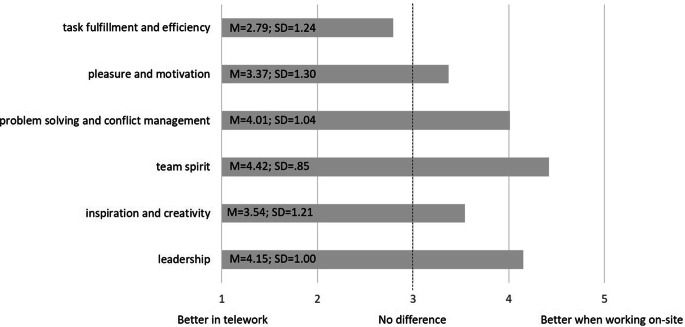
Participants’ ratings for the fulfillment of different qualities of work in telework versus working on-site [*N* = 145] Erfüllung von Arbeitsqualitäten in Telearbeit vs. Vor-Ort-Arbeit

Fig. 2 shows participants’ quantitative ratings for different qualities of work and whether these qualities are better supported through telework or working on site. Participants see digital collaboration as more advantageous than working on-site when it comes to task fulfillment and efficiency. The deviation from the scale midpoint was significant (t (144) = 2.08, *p* < 0.05, d = 0.17). In contrast, working on-site was considered more advantageous than telework for all other surveyed qualities, including pleasure and motivation (t (144) = 3.45, *p* < 0.01, d = 0.28), problem-solving and conflict management (t (144) = 11.73, *p* < 0.001, d = 0.97), leadership (t (144) = 13.93, *p* < 0.001, d = 1.16), team spirit (t (144) = 20.20, *p* < 0.001, d = 1.68), as well as inspiration and creativity (t (144) = 5.41, *p* < 0.001, d = 0.45).

### RQ 2—Social norms

Regarding our second set of research questions, i.e., the relevance of social norms and diverging interpretations of one’s conversation partner’s behavior in conflicts arising in teleworking communication, the conflict reports presented above indicate social norm conflicts are a relevant issue. More than 40% of conflicts referred to a lack of rules, norm violations, or uncertainty, misunderstandings, and lack of social feedback in telework communication, of which thereof 19% explicitly referred to norm violations or a lack of rules. Technical problems remained the most frequently mentioned conflict category, at 32%. However, while the problem itself and potential solutions are more obvious when it comes to technical problems, the described social conflicts are more easily overseen and often only evident to one communication partner, which may lead to lingering conflicts with long-term destructive potential. Regarding perceived challenges of the working-from-home situation, the most frequently mentioned category was isolation and a lack of social interaction. This highlights the relevance of teleworkers’ social needs and the challenges associated with fulfilling these through digital channels, particularly with respect to the feeling of relatedness resulting from small talk and casual conversation. In light of these reduced opportunities, it becomes even more important that interaction with colleagues is as fulfilling and conflict-free as possible.

Thus, as an interim conclusion regarding RQ 2, the vivid descriptions of social conflicts and experiences of others’ norm violations show that these are subjectively relevant for teleworkers and that this issue deserves attention. The social norms perspective can be confirmed as a helpful theoretical frame that provides a more nuanced understanding beyond mere conflict descriptions. First, it impressively indicates *why a behavior represents a conflict*. It explains how conflict-generating behaviors that seem minor on the surface can have strong emotional consequences if such behavior is understood as an act against the group and its basis of cohesion, i.e., shared social norms. For example, in the case of the two whispering colleagues mentioned above, we do not know whether they were actually gossiping about the others or whether this was just irrelevant, mindless behavior. Still, their behavior is understood as an act against social norms and threatens the team climate. Second, the social norms perspective highlights *why it is important to take these conflicts seriously*. Given the association of social norms with basic values and team spirit, social norm conflicts should not be considered isolated episodes between two (or more) communication partners, but instead represent broader, more general threats.

**Fig. 3:**
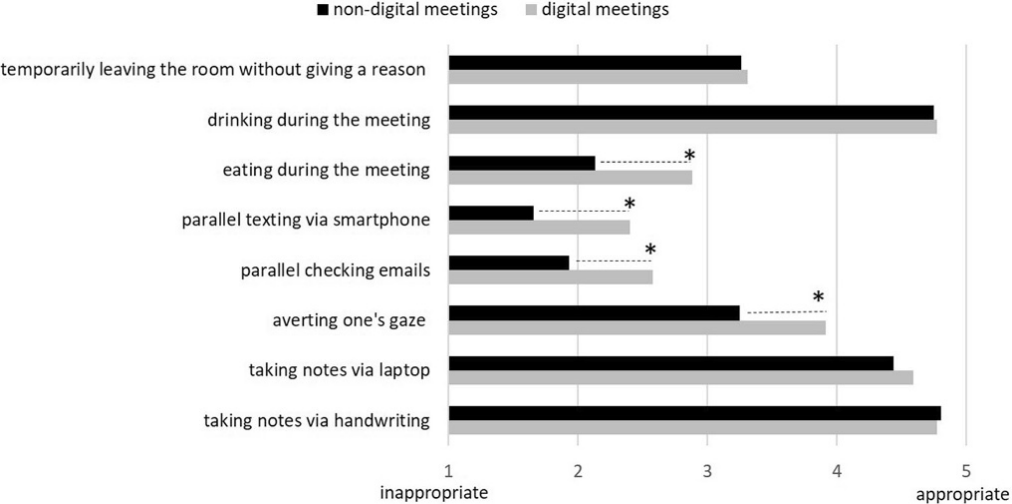
Participants’ ratings of the appropriateness of different behaviors in digital and non-digital meetings [*N* = 145]. (Significant differences between digital and non-digital meetings are marked with an *asterisk*) Soziale Normen in digitalen und nicht-digitalen Meetings

In addition to this analysis of the general relevance of social norms within the reported conflicts, we analyzed ratings of concrete behaviors in digital and non-digital meetings to obtain insight into existing norms and possibly diverging views on appropriate behavior in teleworking communication. Table 7 displays participants’ quantitative ratings of the appropriateness of different behaviors in digital and non-digital meetings. Fig. 3 presents these ratings in a bar graph for easier comparison. In general, the two ratings of each behavior were in the same direction, i.e., the behaviors were rated as either inappropriate (e.g., eating during a meeting) or appropriate (e.g., drinking during a meeting) regardless of the digital or non-digital context. However, a significant difference between the two contexts was found for some behaviors, which were generally seen as more inappropriate in non-digital contexts. For example, eating, parallel texting via one’s smartphone, checking emails or averting one’s gaze from the screen/others was considered rude in both contexts, but relatively more acceptable in digital meetings. One possible reason for the more relaxed attitude towards such behaviors in digital contexts is that the relevant behavior (e.g., texting via smartphone) is less visible to others (e.g., because others cannot see your hands). Of course, the fact that others cannot see a side activity does not eliminate the resulting attention deficits, but at least the behavior may appear less provocative and less disrespectful.

**Table 7:** Participants’ ratings of the appropriateness (1 = inappropriate, 5 = appropriate) of different behaviors in digital and non-digital meetings [*N* = 145] Beurteilung der Angemessenheit (1 = unangemessen, 5 = angemessen) verschiedener Verhaltensweisen in digitalen und nicht-digitalen Meetings [*N* = 145]

Behavior	Digital meeting		Non-digital meeting				
	M	SD	M	SD	T	df	p
Temporarily leaving the room without giving a reason	3.31	1.41	3.26	1.47	0.39	144	0.700
Drinking during the meeting	4.77	0.55	4.76	0.54	0.34	144	0.733
Eating during the meeting	2.88	1.33	2.13	1.14	6.97	144	< 0.001
Parallel texting via smartphone	2.40	1.63	1.67	0.95	7.50	144	< 0.001
Parallel checking emails	2.59	1.20	1.93	1.03	6.59	144	< 0.001
Averting one’s gaze (from the screen/from others)	3.91	1.08	3.25	1.15	6.09	144	< 0.001
Taking notes on a laptop	4.59	0.76	4.41	0.91	2.05	144	0.042
Taking notes by hand	4.78	0.59	4.82	0.51	1.00	144	0.319

**Fig. 4:**
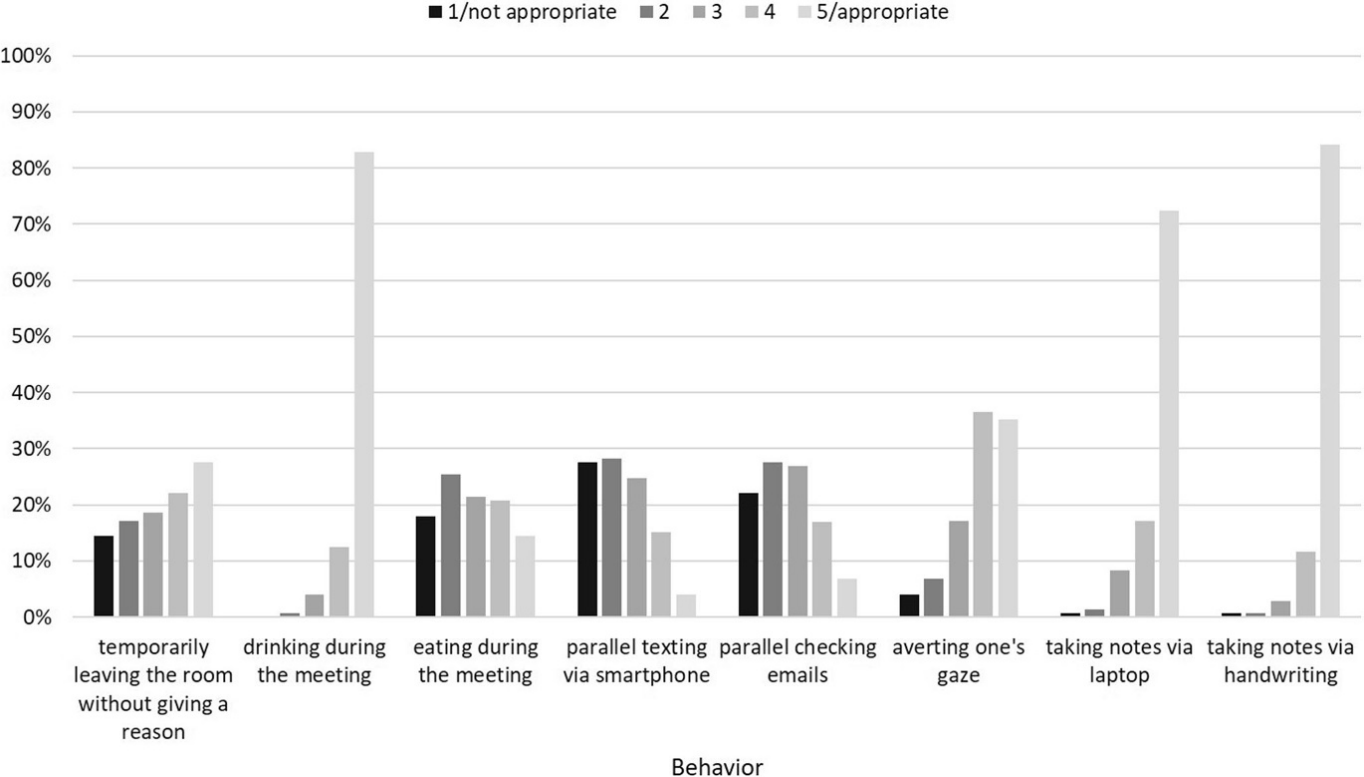
Frequencies of appropriateness ratings for different behaviors in digital meetings [*N* = 145] Häufigkeit der Beurteilung der Angemessenheit verschiedener Verhaltensweisen in digitalen Meetings

Moreover, the distribution of participants’ answers across the five-point rating scale reveals for which behaviors relatively clear norms exist and for which diverging views are present. Fig. 4 shows the distributions of appropriateness ratings for different behaviors in digital meetings. It shows that for some behaviors, such as drinking or taking notes during a meeting, relatively clear views exist, indicating a commonly shared norm. For other behaviors, such as temporarily leaving the room or eating during the meeting, the answers are distributed across the full range of the rating scale, in some cases almost evenly. Such diverging views, with a wide diffusion of answers across the rating scale, are more likely to induce norm conflicts, because a behavior that seems appropriate to one person is regarded as inacceptable by others.

### RQ 3—Interventions and technology design

Regarding the question of helpful interventions to prevent digital communication conflicts in the work context, on the applied quantitative rating scale, all four suggested starting points (i.e., leadership, meta-communication, personal responsibility, technology design) were rated as generally promising and significantly higher than the scale midpoint (= 3), with the highest ratings for meta-communication (e.g., discussing communication rules as a team, M = 4.00, SD = 1.08; t (144) = 11.15, *p* < 0.001, d = 0.93) and technology design (e.g., the technology itself supports commitments to basic team rules such as punctuality, M = 3.99, SD = 1.07; t (144) = 11.13, *p* < 0.001, d = 0.92). In comparison, the ratings for interventions by leadership (e.g., setting clear rules for digital meetings, M = 3.88, SD = 1.11; t (144) = 9.49, *p* < 0.001, d = 0.79) and personal responsibility (e.g., guidelines to support self-reflection in digital meetings, M = 3.62, SD = 1.05; t (144) = 7.09, *p* < 0.001, d = 0.59) were slightly lower. In addition, participants’ qualitative statements provided concrete examples of how to utilize the different starting points. For example, regarding meta-communication, participants’ qualitative statements provided a variety of examples of possible aspects that could be addressed in meta-communication about digital meeting etiquette within teams. These included rules for the order of speakers, agreement about everybody switching their camera on (or off), agreement about muting one’s microphone when not speaking, or assigning two moderators to each meeting (one to lead the conversation, one to operate the software/chat). Regarding technology design, participants’ qualitative statements mostly emphasized the importance of usability and an intuitive user interface design, so that all communication partners are aware of the technical possibilities to transfer communication rules from non-digital meetings to the digital space (e.g., how to use the raise hand feature in Zoom). Some participants also declared that there simply was no way to resolve the described conflict in the context of a digital meeting and that the described conflict could have only been prevented through a face-to-face meeting.

**Fig. 5:**
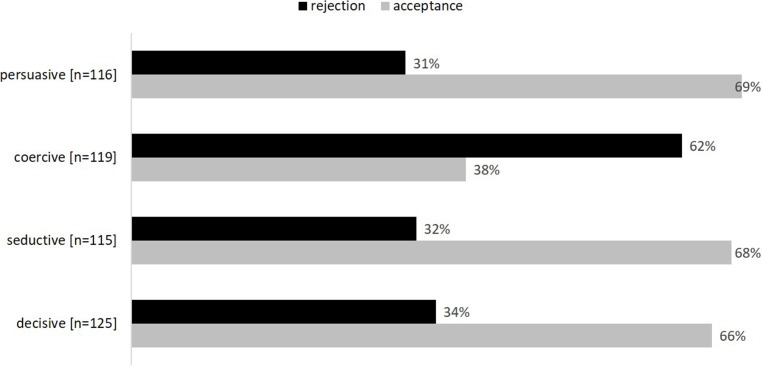
Frequencies of acceptance of different design influence approaches Akzeptanz verschiedener Ansätze der Beeinflussung durch Gestaltung

Finally, we analyzed participants’ ratings of different types of design influence according to the classification scheme by Tromp et al. (2011) for the provided example of how a technology could support punctuality in digital meetings. Table 8 shows the level of agreement on the applied quantitative rating scale for the different types of design influence. Of the four types (persuasive, coercive, seductive, decisive), three strategies gained positive average ratings significantly higher than the scale midpoint, namely persuasive (M = 3.52; SD = 1.33; t (144) = 4.69, *p* < 0.001, d = 0.39), seductive (M = 3.34; SD = 1.29; t (144) = 3.26, *p* < 0.001, d = 0.27), and decisive influence (M = 3.41; SD = 1.50; t (144) = 3.72, *p* < 0.001, d = 0.31). Only the coercive approach received a negative mean rating, significantly below the scale midpoint (M = 2.54; SD = 1.41; t (144) = 3.95, *p* < 0.001, d = 0.33). In addition, to obtain a broad comparative overview of acceptance of the four approaches, we dichotomized participants’ ratings on the five-point scale into acceptance (ratings > 3) and rejection (ratings < 3). Note that participants who gave a rating of 3 were not considered for the dichotomization into acceptance or rejection, resulting in somewhat limited sample sizes. Fig. 5 displays the relative frequencies of acceptance and rejection for each approach. While a coercive videoconferencing system where it is no longer possible to dial in when more than two minutes late was rejected by the majority of participants, the other approaches supporting punctuality through a notification of how long the others are already waiting when dialing in (persuasive), displaying each participant’s time of entry (seductive), or muting participants who are late for the first two minutes to not disrupt the current flow of communication (decisive) were welcomed by the majority of participants.

**Table 8:** Agreement ratings for four different design influence approaches according to the classification scheme by Tromp et al. (2011) and provided examples with respect to supporting punctuality in digital meetings [*N* = 145, 1 = do not agree at all, 5 = totally agree] Zustimmung zu vier verschiedenen Ansätzen der Beeinflussung durch Gestaltung gemäß dem Klassifikationsschema von Tromp et al. (2011) und Umsetzungsbeispiele zur Unterstützung von Pünktlichkeit in digitalen Meetings [*N* = 145, 1 = stimme gar nicht zu, 5 = stimme voll zu]

Type of design influence	Statement + example:	Agreement	
	Technology should be designed in a way that	M	SD
Apparent, weak = persuasive influence	… encourages commitment to rules such as timelines, e.g., a videoconferencing system which encourages timelines by a notification how long the others are already waiting when dialing in	3.52	1.33
Apparent, strong = coercive influence	… enforces commitment to rules such as punctuality, e.g., a videoconferencing system where it is no longer possible to dial in when more than two minutes late	2.54	1.41
Hidden, weak = seductive influence	… supports commitment to rules such as punctuality in a gentle way, e.g., a videoconferencing system that displays each participant’s time of entry	3.34	1.29
Hidden, strong = decisive influence	… supports commitment to rules such as punctuality in a decisive way, e.g., a videoconferencing system that mutes participants who are late for the first two minutes, to not disrupt the current flow of communication	3.41	1.50

## Discussion

In summary, the present findings tell a complex story about teleworkers’ daily experiences and related practical and psychological challenges. Both individual teleworkers as well as team managers responsible for creating a context that facilitates digital interaction within and between teams face high demands. The study shows that successful telework requires a holistic perspective: While creating functional and technical structures that allow people to work and communicate with one another is an important basis, further consideration must be given to psychological variables and social structures. The following sections further discuss our key findings clustered along the predefined research questions and relate these to previous research.

### RQ 1—Conflicts and reflections on telework

The present survey of the everyday experiences of teleworkers in different industries revealed a variety of typical conflicts in digital communication in the work context, which were associated with a negative affective experience for the person who reported the conflict as well as (presumably) the other involved persons, such as colleagues, customers or supervisors. However, the other person’s experience was rated as less negative than one’s own, which may reflect a feeling of being the true “victim” of the conflict oneself. When people think of conflicts, they tend to intuitively think in terms of dichotomous roles of harm-doers/perpetrators versus victims (e.g., Gray and Wegner 2009; SimanTov-Nachlieli and Shnabel 2014), which bears the risk of further escalation. As described in studies on competitive victimhood (Noor et al. 2012, p. 351), each of the conflicting parties sees it as the other party’s responsibility to initiate actions to end the conflict or apologize for their disrespectful behavior, whereas oneself has the right to feel offended. Thus, if both conflict partners see themselves in the same position and expect the other person to deescalate, an escalating process may ensue, where feelings of distance and possibly aggression grow over time (see also Diefenbach and Ullrich 2018).

While about one-third of the reported conflicts were primarily technological in nature, a large share of the remaining conflicts revealed second-order problems regarding social dynamics in the context of digitally mediated communication, often due to a lack of social feedback or lack of shared rules of appropriate behavior. Moreover, it became clear that such misunderstandings often remain unnoticed, and colleagues may not be aware of such norm conflicts and the poor impression they make on others. Instead, individuals may be overconfident that others will interpret their intentions correctly and overestimate the accuracy of their communication behavior in digital meetings, as also found in previous research on email communication (Riordan and Trichtinger 2017). In fact, many of the deficits and communication barriers in digital structures described in this study (e.g., determining who is speaking if you do not know the other participants’ voices very well; feeling less connected than in direct meetings; little room for informal talk) paralleled those already reported in previous research (e.g., Biehl et al. 2015; Egido 1988; Olson and Olson 2000). As already discussed in the related work section, early analyses of the potential and failures of videoconferencing showed that reduced opportunities for informal, unofficial interactions make teleconferencing unattractive and thus in many cases a poor substitute for face-to-face meetings (Egido 1988). Especially when it comes to strategic decisions of long-term relevance, important information transmission tends to happen outside of official meeting rooms (Mintzberg 1973) whereas a videoconference is always “officially” planned and consciously established, people do not dial in by chance.

One might even ask why, despite the well-known limitations of videoconferencing and related remote communication techniques, telework has expanded so much and been considered the work model of the future, even before the pandemic (IWG 2018, 2019). Indeed, how telework stacks up critically depends on the criterion and qualities of work qualities examined. While participants saw working on-site as advantageous with regards to many qualities of work of a social or creative character (e.g., team spirit, problem-solving and conflict management, inspiration and creativity), they saw telework as advantageous for task fulfillment and efficiency. This parallels findings from earlier studies revealing lower satisfaction with collaboration with colleagues among teleworkers (Iometrics and Global Workplace Analytics 2020) but higher self-perceived productivity due to fewer interruptions (Baruch 2000). From a managerial perspective, it is understandable that gains in efficiency and cost reduction (e.g., due to limited travelling, fewer workspaces) are a good argument for telework. However, how qualities of work such as team spirit or creative atmosphere can be supported in the telework context should be further examined. In this regard, the social norms perspective and issues discussed with respect to RQ2 and RQ3 offer some interesting starting points, as discussed in the next sections. In general, if a choice is possible, team managers are well-advised to consider which meetings can be performed digitally without any reduction in quality or even benefit from digital structures—and when meeting in person is required.

Apart from the communication tools used, another aspect of telework concerns the pandemic-induced working-from-home situation. As shown by the participants’ reports, many appreciate the enhanced autonomy and possibilities to integrate work tasks with activities that enhance personal wellbeing, such as cooking healthy meals or taking a short walk around the block in between meetings. At the same time, the participants’ responses emphasized challenges related to the working-from-home situation. Aside from pragmatic issues likely related to the sudden shift to working from to home office in the pandemic context (e.g., not having the necessary technical equipment, non-ergonomic furniture), participants described a number of psychological challenges, which can be broadly differentiated into self-related and other-related challenges. Other-related challenges refer to feelings of loneliness and a lack of companionship due to limited interactions with colleagues, whereas self-related challenges are linked to increased self-management requirements when working from home, encompassing the categories of boundary management, distractions/concentration problems, and motivation and self-regulation issues. Such reports are in line with previous studies of telework, which have also emphasized difficulties maintaining close relationships with co-workers, a lack of team spirit and motivation, and difficulties in self-organization (Raišienė et al. 2020), self-regulation and boundary management (e.g., Beigi et al. 2018; Iometrics & Global Workplace Analytics 2020). For each of these aspects, more general theories of work-related wellbeing can be considered in order to identify potential starting points for interventions. For example, this is the case regarding boundary management. Boundary theory claims that people create and attempt to maintain psychological and physical behavioral boundaries between different life domains, in line with their individual preference to either separate these life domains from each other or integrate them in order to fulfill specific roles (Ashforth et al. 2000; Olson-Buchanan and Boswell 2006). Naturally, working from home, where a physical boundary between the office and one’s living space no longer exists, poses additional challenges for boundary creation. Thus, individuals might experience the pandemic-induced working-from-home situation as more or less challenging depending on a combination of contextual factors (e.g., having young children at home, living situation), individual preferences, and individual psychological skills (e.g., capacities of self-control and translating intentions into action, Bieleke and Keller 2021; Gollwitzer and Sheeran 2006). Accordingly, the 2020 Global Work-from-Home Experience Survey conducted by Iometrics and Global Workplace Analytics (2020) also identified individual abilities, such as self-regulation, as the most important predictor of working from home successfully, while highlighting boundary management training for employees as an important workplace intervention in response to the pandemic (Chang et al. 2021).

To summarize, these findings clearly indicate the need for specific supports regarding not only the technical but also the “psychological equipment” necessary for telework. Especially if the shift to telework comes abruptly, employees may feel that they are equipped with the technology allowing them to work from home—but no prescription of how to successfully integrate telework with their psychological needs and private life. Therefore, in addition to obligatory training when a new software is introduced, employees should also receive training in psychological competencies such as self-regulation and boundary management, which is becoming all the more necessary in light of the expansion of telework. In addition, required competencies on a team level grow as well, with the development of shared social norm in the digital space being one aspect, as discussed in the next section (RQ2).

### RQ 2—Social norms and norm conflicts

When it comes to communication via digital structures in the context of telework, our survey revealed social norms and misunderstandings due to limited communication cues as a main source of conflict, but also highlighted technology design as a promising solution to address at least some of these issues. As already widely discussed in the literature on organizational citizenship behavior (OCB) and leadership (e.g., Ehrhart and Naumann 2004; Ellemers et al. 2004), social norms and shared rules of interaction are closely connected to social team identity and work motivation—in short, a valuable good to be protected, albeit one that is dependent on the behavior of many. Accordingly, Opp (Hechter and Opp 2001; p. XV) describes social norms as second-order public goods that are most likely to emerge when they are instrumental for the attainment of group goals. However, in contrast to communication breakdowns related to technical problems (e.g., internet connection issues), communication breakdowns related to norm conflicts and resulting bitterness in a team may remain unnoticed, making them even more dangerous.

In particular, team members may see themselves in the morally superior position (and the other person in the wrong) when no clear norms exist. Indeed, our survey showed that for some behaviors in digital meetings, appropriateness ratings were quite diverse, indicating that no clear norms have been established yet. For example, while there was a uniform view that drinking during a meeting is appropriate, participants had divided opinions on eating during meetings: About 45% see it as inappropriate, 35% see it as appropriate, with the remaining 20% selecting the scale midpoint, possibly because they were undecided. A similarly split opinion occurred for checking emails in parallel or temporarily leaving the room without giving a reason. It should be noted that some caution is warranted in interpreting these findings. We do not know whether all participants interpreted the described behaviors in the exact same way, which could be another source of the divergent ratings. In addition, there are some overlaps between the behavioral categories. For example, “parallel texting via smartphone” overlaps with “averting one’s gaze”, which makes it hard to tell which aspect is the more important norm violation. Still, in combination with the many qualitative reports on conflicts related to a lack of rules and norm violations, we can conclude that some potential for conflict exists. Hence, in order to maintain a healthy team climate, it seems necessary to establish shared views on respectful behavior in digital (and non-digital) collaboration within the team. While managing social dynamics in meetings and creating a healthy communication culture is challenging for all organizations, it becomes even more difficult in telework. However, one might also consider the digital context as an opportunity and ask how technology could assist with aspects beyond mere information transmission, as explored in RQ3 and discussed in the next paragraphs.

### RQ 3—Interventions and technology design

In our survey, many conflicts were associated with a lack of communication rule and norms, and participants strongly agreed with the idea of technology being designed in a way that actively facilitates commitment to basic social rules. Still, before implementing seemingly helpful features to support norm commitment, a sensible definition of the exact design goals to be pursued and a thorough understanding of the potential conflict being designed for are necessary for responsible technology design. As discussed in previous work (Diefenbach and Ullrich 2018, p. 53), there is a widespread tendency to constantly invent new features and technological “solutions”, which might later turn out to be social problems. “Read” receipt features in messenger apps, which result in pressure to give an immediate and thus often rushed and ambiguous answer, are just one example of unwanted side effects. Consequently, users sometimes search for workarounds to deactivate such features, such as reading others’ messages with their phone in airplane mode (Griffiths 2016).

With respect to design interventions, different types of norm conflicts identified in earlier research could be valuable starting points. For example, Diefenbach and Ullrich (2018) describe four distinct types of typical norm conflicts in digital spaces, one of which is called “norm confusion”. Norm confusion emerges if technology opens up new fields of social interaction where no previous experience and no clear norms exist yet. These authors note that people may try to find parallels and apply norms from similar situations in the non-digital space, but may not always see an unambiguous parallel: “Which norms, for example, should apply for chat conversations? Should one consider it a personal face-to-face conversation, thus having to say goodbye before leaving the (chat) room? Or is it a non-committal open channel, an occasional meeting zone such as a marketplace, where people are strolling around, may exchange a few words with one person or the other, without a felt obligation to say goodbye to everybody before leaving the zone” (Diefenbach and Ullrich 2018, p. 45). Accordingly, in cases of norm confusion, one could think of design approaches that activate an existing norm and thus support a shared understanding of behavioral rules in a given context. To name a simple example with respect to chat conversation: if a chat is designed to look more like a “real” face-to-face conversation, this may activate a norm of greeting and farewell. Textboxes that look like speech bubbles and photos or avatars to represent the communication partners could be simple starting points (see also Mooseder 2018).

Another approach could be to support self-monitoring in digital contexts. In many of the reported norm conflicts, participants complained about others’ limited awareness of the impression they made on others. In general, users do not reflect on their own behavior to the same extent observers do (e.g., answering a phone call in public and speaking way too loudly, Diefenbach and Ullrich 2018, p. 54), and a failure to apply social norms can be related to a lack of self-control (Voggeser et al. 2018). In videoconferencing, for example, simple statistics about one’s communication behavior such as talking time, frequency of interrupting others, or indicators of background noise could support self-monitoring and help to bridge discrepancies between communication partners to some degree.

## Conclusion

Digital communication channels have proven their potential as a way to collaborate with colleagues and customers during the rapid expansion of telework in response to the Covid-19 pandemic probably more than even before. In conclusion, the present survey yielded an encouraging perspective on telework overall, but also pointed out areas that require further attention. More specifically, it confirmed that autonomy, flexible time management and related wellbeing improvements constitute core benefits of telework for employees. However, it also became clear that certain qualities, such as creativity and informal interaction, develop less naturally in digital contexts and require specific support. In addition, our study highlighted typical conflicts related to telework. In many cases, these reflected conflicts around social norms or a lack of social feedback due to the inherent limitations of digital channels. In addition to meta-communication about shared rules of behavior in teams, the design of technologies themselves could be a starting point to reduce conflict. Thereby, this research emphasizes the potential of technology design to improve digital collaboration beyond information exchange and highlights communication qualities from a psychological perspective. Extending previous studies on wellbeing in relation to telework, it places particular emphasis on the social dynamics behind conflicts and perceived challenges and highlights social norms as a useful frame that may also be addressed in design decisions.

It should be noted that the present findings reflect the experiences of a limited sample of teleworkers, collected in one country, and therefore need to be interpreted with caution. Still, while the exact mechanisms behind the revealed conflicts, theoretical explanations and transferability to other samples require further examination, the present research clearly highlights the necessity to address psychological wellbeing in teleworking scenarios and to use insights into teleworkers’ subjective experiences to inform technology design. While the creation of digital structures and relevant software is an important prerequisite for successful teleworking, specific psychological challenges, effects on psychological wellbeing and social dynamics also require attention. This pertains particularly to subtle, less obvious effects, which do not arouse strong emotions at first glance but may disrupt social structures in the long run. By shifting these issues into focus, the present study seeks to provide a basis for a successful and wellbeing-oriented future for telework.

## References

[CR1] Abendroth AK, Reimann M (2018). Telework and work-family conflict across workplaces: Investigating the implications of work-family-supportive and high-demand workplace cultures. Contemporary perspectives in family research: The work-family interface: Spillover, complications, and challenges.

[CR2] Allen TD, Golden TD, Shockley KM (2015). How effective is telecommuting? Assessing the status of our scientific findings. Psychol Sci Public Interest.

[CR3] Ars Technica Open Forum (2014) How do you address someone in your reply to their emails? https://arstechnica.com/civis/viewtopic.php?f=23&t=1244027. Accessed 7 Feb 2021

[CR4] Ashforth BE, Kreiner GE, Fugate M (2000). All in a day’s work: Boundaries and micro role transitions. Acad Manag Rev.

[CR5] Baruch Y (2000). Teleworking: benefits and pitfalls as perceived by professionals and managers. New Technol Work Employ.

[CR6] Beigi M, Shirmohammadi M, Stewart J (2018). Flexible work arrangements and work–family conflict: A metasynthesis of qualitative studies among academics. Hum Resour Dev Rev.

[CR7] Biehl JT, Avrahami D, Dunnigan A (2015). Not really there: Understanding embodied communication affordances in team perception and participation. Proceedings of the 18th ACM Conference on Computer Supported Cooperative Work & Social Computing.

[CR8] Bieleke M, Keller L (2021). Individual differences in if-then planning: Insights from the development and application of the If-Then Planning Scale (ITPS). Pers Individ Dif.

[CR9] Browne D (2020) Along with virtual learning, lessons in online etiquette. https://leader.pubs.asha.org/do/10.1044/leader.SCM.25102020.38/full/. Accessed 28 July 2021

[CR10] Cambridge Dictionary. https://dictionary.cambridge.org/de/worterbuch/englisch/telework. Accessed 1 Feb 2021

[CR11] Campion M, Thayer P (1987). Job design: Approaches, outcomes, and trade-offs. Organ Dyn.

[CR13] Chang CH, Shao R, Wang M, Baker N (2021). Workplace interventions in response to COVID-19: an occupational health psychology perspective. Occup Health Sci.

[CR14] Clark HH (1996). Using language.

[CR80] Diefenbach S, Hassenzahl M (2019). Combining model-based analysis with phenomenological insight: A case study on hedonic product quality.. Qualitative Psychology.

[CR15] Diefenbach S, Ullrich D (2018). Disrespectful technologies. Social norm conflicts in digital worlds. Proceedings of the AHFE 2018 International Conferences on Usability & User Experience and Human Factors and Assistive Technology.

[CR16] Diefenbach S, Ullrich S, Kronseder M, Stockkamp M, Weber M, Hess S, Fischer H (2017). (Dis)Respectful Technologies: Wie Technik soziale Normen aushebelt – und was sich dagegen tun lässt. Mensch und Computer 2017 – Usability Professionals 2017.

[CR17] Dixon M (2018) Phone interview with CNBC. https://www.cnbc.com/2018/05/30/70-percent-of-people-globally-work-remotely-at-least-once-a-week-iwg-study.html. Accessed 1 Feb 2021

[CR18] Driska AP, Reid MA, Coumbe-Lilley JE, Shipherd AM (2020). Creating a community of learners in online courses. High impact teaching for sport and exercise psychology educators.

[CR19] Egido C (1988). Video conferencing as a technology to support group work: a review of its failures. Proceedings of the 1988 ACM conference on Computer-supported cooperative work.

[CR21] Ehrhart MG, Naumann SE (2004). Organizational citizenship behavior in work groups: a group norms approach. J Appl Psychol.

[CR20] Ellemers N, De Gilder D, Haslam SA (2004). Motivating individuals and groups at work: A social identity perspective on leadership and group performance. Acad Manage Rev.

[CR22] Fogg BJ (2003). Persuasive technologies. Using computers to change what we think and do.

[CR23] Gajendran RS, Harrison DA (2007). The good, the bad, and the unknown about telecommuting: meta-analysis of psychological mediators and individual consequences. J Appl Psychol.

[CR27] Gollwitzer PM, Sheeran P (2006). Implementation intentions and goal achievement: A meta-analysis of effects and processes. Adv Exp Soc Psychol.

[CR24] Gray K, Wegner DM (2009). Moral typecasting: Divergent perceptions of moral agents and moral patients. J Pers Soc Psychol.

[CR25] Griffiths S (2016) How to read WhatsApp messages WITHOUT your friends knowing: Simple trick uses Airplane Mode to stop ‘blue ticks’ appearing. Daily Mail online, 16/01/2016. https://www.dailymail.co.uk/sciencetech/article-3410133/How-read-WhatsApp-messages-without-friends-knowing-Simple-trick-uses-Airplane-Mode-stop-blue-ticks-appearing.html. Accessed 11 Feb 2021

[CR29] Hechter M, Opp K-D (2001). Social norms.

[CR30] Hemmerich W (2015) StatistikGuru: Cohen’s d berechnen. https://statistikguru.de/rechner/cohens-d.html. Accessed 13 Oct 2022

[CR31] Hemmerich W (2020) StatistikGuru: Cohen’s d für den gepaarten t‑Test berechnen. https://statistikguru.de/rechner/cohens-d-gepaarter-t-test.html. Accessed 13 Oct 2022

[CR32] Herzberg F (1974). Motivation-hygiene profiles: Pinpointing what ails the organization. Organ Dyn.

[CR33] Hong FY, Lin CC, Lin TJ, Huang DH (2019). The relationship among the social norms of college students, and their interpersonal relationships, smartphone use, and smartphone addiction. Behav Inf Technol.

[CR34] Horne C, Hechter M, Opp K-D (2001). Sociological perspectives on the emergence of social norms. Social norms.

[CR35] Inbar O, Joost G, Hemmert F, Porat T, Tractinsky N (2014). Tactful calling: investigating asymmetric social dilemmas in mobile communications. Behav Inf Technol.

[CR36] Iometrics & Global Workplace Analytics (2020) Global work-from-home experience survey. https://globalworkplaceanalytics.com/whitepapers. Accessed 1 Feb 2021

[CR37] Isaacs E, Tang JC (1993). What video can and can’t do for collaboration: A case study. Proceedings of the First ACM International Conference on Multimedia.

[CR38] IWG International Workplace Group (2018) The workspace revolution: reaching the tipping point. Global insights on flexible working from 18,000 professionals in 96 countries. http://bpcc.org.pl/uploads/ckeditor/attachments/14371/IWG_report.pdf. Accessed 17 Jan 2021

[CR39] IWG International Workplace Group (2019) The IWG global workspace survey. Welcome to Generation Flex—the employee power shift. https://assets.regus.com/pdfs/iwg-workplace-survey/iwg-workplace-survey-2019.pdf. Accessed 17 Jan 2021

[CR41] Kim AJ (2000). Community building on the web.

[CR42] Laschke M, Hassenzahl M, Diefenbach S (2011). Things with attitude: Transformational products. Proceedings of Create11 conference.

[CR43] Lee MK, Takayama L (2011). Now, I have a body: Uses and social norms for mobile remote presence in the workplace. Proceedings of the SIGCHI Conference on Human Factors in Computing Systems.

[CR44] Lockton D, Nicholson L, Cain R, Harrison D (2014). Persuasive technology for sustainable workplaces. Interactions.

[CR45] Mann S, Holdsworth L (2003). The psychological impact of teleworking: stress, emotions and health. New Technol Work Employ.

[CR46] Martey RM, Stromer-Galley J (2007). The digital dollhouse: context and social norms in the Sims Online. Games Cult.

[CR48] Messenger JC (2019). Telework in the 21st century: an evolutionary perspective.

[CR49] Milasi S, González-Vázquez I, Fernández-Macías E (2020) Telework in the EU before and after the COVID-10: where we were, where we head to. Report by the European Commissions’s science and knowledge service Joint Research Centre. https://ec.europa.eu/jrc/sites/jrcsh/files/jrc120945_policy_brief_-_covid_and_telework_final.pdf. Accessed 1 Feb 2021

[CR50] Miller HJ, Thebault-Spieker J, Chang S, Johnson I, Terveen L, Hecht B (2016). “Blissfully happy” or “ready to fight”: varying interpretations of Emoji. Proceedings of the 10th International AAAI conference on Web and Social Media (ICWSM 2016).

[CR51] Mintzberg H (1973). The nature of managerial work.

[CR52] Moncur W, Orzech KM, Neville FG (2016). Fraping, social norms and online representations of self. Comput Human Behav.

[CR53] Mooseder A (2018). The impact of the design of communication media on the activation of social norms [Einfluss der Gestaltung von Kommunikationsmedien auf die Aktivierung sozialer Normen]. Unpublished Bachelors Thesis, Department of Psychology, Ludwig-Maximilians-Universität München, Munich, Germany

[CR54] Nkwo MS (2019). Designing culturally-appropriate persuasive technology to promote positive work attitudes among workers in public workplaces. Proceedings of the 27th ACM Conference on User Modeling, Adaptation and Personalization (UMAP’19).

[CR2012] Noor M, Shnabel N, Halabi S, Nadler A (2012). When suffering begets suffering: The psychology of competitive victimhood between adversarial groups in violent conflicts. Pers Soc Psychol Rev.

[CR55] Olson GM, Olson JS (2000). Distance matters. Hum Comput Interact.

[CR56] Olson-Buchanan JB, Boswell WR (2006). Blurring boundaries: Correlates of integration and segmentation between work and nonwork. J Vocat Behav.

[CR58] Pejsa T, Kantor J, Benko H, Ofek E, Wilson A (2016). Room2room: Enabling life-size telepresence in a projected augmented reality environment. Proceedings of the 19th ACM Conference on Computer-Supported Cooperative Work & Social Computing.

[CR59] Popa M, Phillips BJ, Robertson C (2014). Positive outcomes of social norm transgressions. J Consum Behav.

[CR60] Preece J (2004). tiquette online: From nice to necessary. Commun ACM.

[CR61] Radford ML (2006). Encountering virtual users: A qualitative investigation of interpersonal communication in chat reference. J Am Soc Inf Sci.

[CR62] Raišienė AG, Rapuano V, Varkulevičiūtė K, Stachová K (2020). Working from Home—Who is Happy? A Survey of Lithuania’s employees during the COVID-19 quarantine period. Sustainability.

[CR63] Remer A (1978). Personalmanagement: Mitarbeiterorientierte Organisation und Führung von Unternehmen.

[CR64] Riordan MA, Trichtinger LA (2017). Overconfidence at the keyboard: Confidence and accuracy in interpreting affect in e-mail exchanges. Hum Commun Res.

[CR65] Sabra JB (2017). “I hate when they do that!” Netiquette in mourning and memorialization among Danish Facebook users. J Broadcast Electron Media.

[CR66] Sánchez AM, Pérez MP, de Luis Carnicer P, Jiménez MJV (2007). Teleworking and workplace flexibility: a study of impact on firm performance. Pers Rev.

[CR67] Sardeshmukh SR, Sharma D, Golden TD (2012). Impact of telework on exhaustion and job engagement: A job demands and job resources model. New Technol Work Employ.

[CR68] Sauer J, Schramme S, Rüttinger B (2000). Knowledge acquisition in ecological product design: the effects of computer-mediated communication and elicitation method. Behav Inf Technol.

[CR69] Schaffner M (2008). Meeting etiquette. Gastroenterol Nurs.

[CR70] Selassie HH, Oyibo K, Vassileva J (2017). Responsiveness to persuasive strategies at the workplace: A case study. Proceedings of the International Conference on E-Technologies.

[CR71] SimanTov-Nachlieli I, Shnabel N (2014). Feeling both victim and perpetrator: Investigating duality within the needs-based model. Pers Soc Psychol Bull.

[CR72] Tene O, Polonetsky J (2014). A theory of creepy: technology, privacy and shifting social norms. Yale J Law Technol.

[CR73] Tromp N, Hekkert P, Verbeek P-P (2011). Design for socially responsible behavior: A classification of influence based on intended user experience. Des Issues.

[CR74] Turner JC (1991). Social influence.

[CR75] Vittersø J, Akselsen S, Evjemo B, Julsrud TE, Yttri B, Bergvik S (2003). Impacts of home-based telework on quality of life for employees and their partners. Quantitative and qualitative results from a European survey. J Happiness Stud.

[CR76] Voggeser BJ, Singh RK, Göritz AS (2018). Self-control in online discussions: Disinhibited online behavior as a failure to recognize social cues. Front Psychol.

[CR77] Whittaker S (1995). Rethinking video as a technology for interpersonal communications: theory and design implications. Int J Hum Comput Stud.

[CR78] Williams-Jones B (2020) Zoom meetings and the need for evolving social norms. https://observatoire-ia.ulaval.ca/zoom-meetings-and-the-need-for-evolving-social-norms/. Accessed 28 July 2021

[CR79] Yee N, Bailenson JN, Urbanek M, Chang F, Merget D (2007). The unbearable likeness of being digital: The persistence of nonverbal social norms in online virtual environments. Cyberpsychol Behav.

